# PBJ ranks higher, enhances diversity and offers free global access

**DOI:** 10.1111/pbi.13528

**Published:** 2020-12-28

**Authors:** Henry Daniell

**Affiliations:** ^1^ School of Dental medicine University of Pennsylvania Philadelphia PA USA

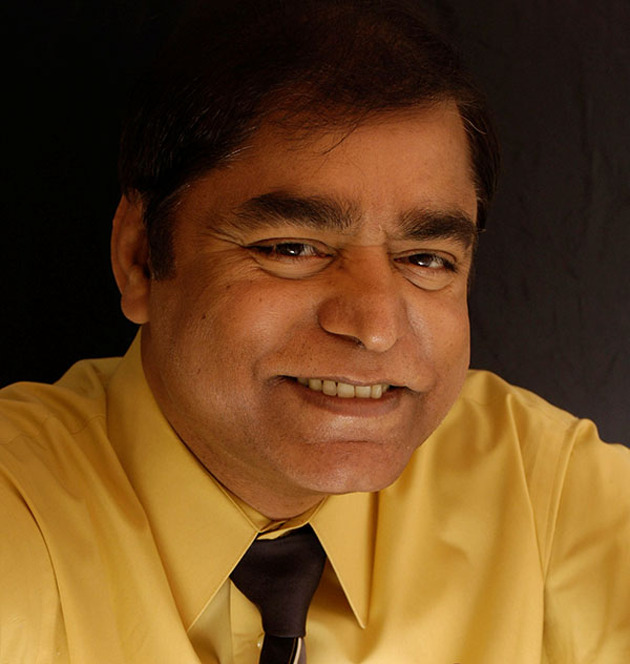

Welcome to this first issue of the nineteenth volume of the *Plant Biotechnology Journal* (PBJ), an open access plant science journal offering free global access to our readers through the open access fee paid by our authors. Amidst COVID‐19 challenges, I thank all our authors, reviewers and editors for their valuable contributions and evaluations to advance PBJ. PBJ accomplishments listed below are the result of continued dedication and hard work of the entire PBJ team.

PBJ increased its Impact Factor from 6.84 to 8.15 this year, ranking higher than much older plant science journals. PBJ maintains #5 IF ranking among plant science journals publishing original research articles. Based on my current evaluation of citations, PBJ is likely to reach an Impact Factor greater than 9.0 next year ‐ thanks to exceptionally high citations, with several articles that are ranked within the top one percentile of articles published in plant and animal sciences. Scopus CiteScore continues to rank PBJ first among 334 Agronomy and Crop Science journals. Excluding review journals in plant sciences, CiteScore ranks PBJ third (14.1), similar to the Plant Cell (14.1) but behind Nature Plants (19.4) and Molecular Plant (14.8). PBJ has the highest cited articles (95%) when compared to Molecular Plant (93%) and Nature Plants (83%). Irrespective of the method of citation analysis, PBJ is building on the strength of high impact original inventions reported by our authors and critically evaluated by our editors/reviewers.

Since I started as the Editor‐in‐Chief in 2012, submission of manuscripts has almost tripled, despite transition to an open access journal a few years ago. Despite COVID‐19, the number of submissions to PBJ continued to increase in 2020. PBJ received manuscripts from 54 countries representing all continents around the globe in 2020, highlighting the breadth of countries that submit to PBJ. I am deeply grateful for thousands of critical evaluations received from reviewers and their generous time commitment. Timely reviews amidst the COVID pandemic have significantly decreased the average turnaround time (although few manuscripts were delayed when reviewers/editors experienced COVID‐19), rewarding our authors with timely decisions on their submissions. Considering PBJ is still a very young plant science and biotechnology journal, these are very impressive accomplishments.

PBJ has increased social media activities in 2020, with the help of Professor Shuangxia Jin (PBJ Associate Editor, Huazhong Agricultural University, China) launching the PBJ WeChat account (PBJ ID: PBJ201903) on March 1, 2019. In 2020, this WeChat account has published 2392 news articles including 453 original articles written by his students and 1939 articles cited from other social media sources. This has resulted in 34 700 plant scientists' WeChat follower accounts, with 3 073 457 hits from 1 722 590 computers, 8400 hits/day and 2500–3000 hits per news of each publication from *Plant Biotechnology Journal*. I now request that authors provide Twitter messages at the time of manuscript submission. Posts from the PBJ Twitter account @PlantBiotechJ have generated an average daily rate of 120 tweet impressions, for a total a 43 500 impressions over the last 12 months. In addition, I encourage authors to share news releases on their articles with the PBJ editorial office, so that they can be included in Wiley Plant Science tweets @wileyplantsci, which currently has >16 900 followers. Readership of PBJ (i.e. full‐text downloads or articles) has also increased significantly over the last few years to over 1.2 million downloads in 2020.

I convey my sincere thanks for the excellent service provided by Senior Editor Prof. Dominique Michaud, all our Associate Editors (Profs. François Belzile, Xiao‐Ya Chen, Dave Edwards, Zuhua He, Shuangxia Jin, Marco Maccaferri, Johnathan Napier, Martin Parry, Nicola Patron, Stephen Streatfield, Neal Stewart, Rajeev Varshney and Kan Wang) and those who joined PBJ recently (Prof. Anthony Hall, Drs. Thomas Jacobs, Yanfei Mau, Yiping Qi, Leena Tripathy and Bing Yang). PBJ website features brief biographies of all PBJ editors to introduce their research areas to our readers and authors. I convey my special thanks to Prof. Malcolm Campbell for many years of his service to PBJ and Drs. Jihong Liu‐Clarke, Caixia Gao for their recent service to PBJ.

Ethnicity in scientific journals has emerged as a major concern in 2020 globally. PBJ has 10 white/11 non‐white Associate Editors when compared to 12 white/1‐non‐white in Plant Cell, Annual Review of Plant Biology and 13 white/1 non‐white in Plant Physiology. Thus, PBJ has a high diversity of Associate Editors representing different races, geographical locations and gender balance, reflecting authors from American institutions (37 white/62 non‐white corresponding authors and 269 white/403 non‐white co‐authors). PBJ is currently revitalizing the Editorial Board and inviting suggestions for new members to increase diversity further.

Without the outstanding leadership of Ms. Rosie Trice – Senior Publishing Manager at Wiley, Oxford, PBJ would not be able to function and I convey my deepest appreciation. I thank Ms. Maricar Dumlao – PBJ production editor at Wiley, Manila for efficient and timely production and release of PBJ issues every month, Ms. Rajalakshmi Sundararamanujam for continuing production from Chennai, India, Ms. Madhura Amdekar for her expertise in evaluation of image manipulation, Ms. Reshma Raghu for editorial handling of manuscripts and Ms. Lia Curtin (Associate Managing editor).

PBJ offers several new options for readers to evaluate the short and long‐term impact of published articles, including Altmetric scores, article readership and citations. I encourage all readers to visit the journal homepage (https://onlinelibrary.wiley.com/journal/14677652) to take advantage of open access, to keep up to date with the latest developments and to sign up for our automated e‐alerts in order to receive emailed notifications when new issues or Early View articles are published. Please note that readers should ‘opt‐in’ to receive e‐alerts, by visiting the journal homepage and registering at the ‘Get Content Alerts’ area.

PBJ management has approved my request to waive or reduce open access fees for manuscripts recommended for publication from authors who do not have adequate funding for publications. I am fully committed to advancing PBJ's mission of publishing high quality manuscripts with free global access and I look forward to your continued support in 2021.

